# Magnetic resonance arthrography: what is its importance in the present day?

**DOI:** 10.1590/0100-3984.2018.51.2e1

**Published:** 2018

**Authors:** Guinel Hernandez Filho

**Affiliations:** 1 MD, Radiologist for the Grupo Musculoesquelético da Teleimagem/HCor, Grupo Alta/DASA, Attending Physician at Santa Casa de São Paulo, São Paulo, SP, Brazil. E-mail: guinel31@gmail.com

There have been recent advances in the evolution of magnetic resonance imaging (MRI), including the use of 3.0 T machines in clinical practice, as well as improvements in the sequences and protocols employed in the examination of the musculoskeletal system^(1)^. Those advances have given rise to a question: what is the real need for minimally invasive diagnostic procedures, specifically magnetic resonance arthrography (MRA), in the assessment of intra-articular disorders?

The MRA examination has proven to be a diagnostic method of great sensitivity and specificity for the evaluation of conditions that affect intra-articular structures, such as instability of the capsuloligamentous structures of the shoulder, providing a more detailed evaluation of the changes to the articular cartilage surface and glenoid labrum^(2,3)^. In relation to the hip joint, MRA has also been the object of studies of femoroacetabular impingement, in which it has been shown to have excellent sensitivity and specificity for the staging of lesions of the articular cartilage and labrum^(4)^. Even in situations in which conventional MRI can meet the diagnostic needs in a broad sense, such as studies of the knee, MRA can still play a role in specific aspects, such as the postoperative evaluation of meniscal lesions^(5)^. Therefore, it can be said that MRA is still indicated for the diagnosis of some specific intra-articular conditions, including those affecting other joints, such as the elbow, wrist, and ankle.

The MRA procedure requires specific management of the patient, with a larger team of professionals; in most cases, MRA is used in conjunction with another imaging modality (X-ray, ultrasound, or computed tomography) to guide the puncture, as well as requiring the investment of additional time on the part of the radiologist^(6,7)^. Albeit a minimally invasive procedure, MRA is not totally free of complications, which can include infection, reaction to the contrast medium, and post-puncture pain^(8)^. In addition, patients can become anxious when they are informed of the details of the procedure^(9)^, which can therefore be suspended due to non-consent.

Due to the greater complexity of the MRA, there have been various studies comparing conventional MRI and MRA, in terms of their diagnostic efficacy. Although some authors found no significant difference between the two methods^(10)^, the vast majority of such studies demonstrated the superiority of MRA for the evaluation of lesions of the articular cartilages and labra, especially of those in the shoulder and hip^(11,12)^.

Some studies have also helped demystify the topic of patient discomfort during MRA. In an article published in this issue of Radiologia Brasileira, Nascimento et al.^(13)^ concluded, on the basis of the responses obtained on questionnaires applied before and after the MRA examination, that the procedure is less painful than patients expect. The authors also described a novel technique in which the puncture is guided by conventional radiography.

It should be borne in mind that the techniques involved in the use of new MRI equipment and sequences, together with the continuing education and specialization of radiologists, have helped reduce the number of MRA exams. However, there are still evidence-based formal indications for the use of this imaging modality, which can be performed with relative safety and comfort for the patient.
